# Targeted Deletion of p73 in Mice Reveals Its Role in T Cell Development and Lymphomagenesis

**DOI:** 10.1371/journal.pone.0007784

**Published:** 2009-11-11

**Authors:** Alice Nemajerova, Gustavo Palacios, Norma J. Nowak, Sei-ichi Matsui, Oleksi Petrenko

**Affiliations:** 1 Department of Pathology, State University of New York at Stony Brook, Stony Brook, New York, United States of America; 2 Department of Medicine, Albert Einstein College of Medicine, New York, New York, United States of America; 3 Department of Biochemistry and Center of Excellence in Bioinformatics and Life Sciences, University at Buffalo, State University of New York, Buffalo, New York, United States of America; 4 Roswell Park Cancer Institute, Buffalo, New York, United States of America; Roswell Park Cancer Institute, United States of America

## Abstract

Transcriptional silencing of the p73 gene through methylation has been demonstrated in human leukemias and lymphomas. However, the role of p73 in the malignant process remains to be explored. We show here that p73 acts as a T cell-specific tumor suppressor in a genetically defined mouse model, and that concomitant ablation of p53 and p73 predisposes mice to an increased incidence of thymic lymphomas compared to the loss of p53 alone. Our results demonstrate a causal role for loss of p73 in progression of T cell lymphomas to the stage of aggressive, disseminated disease. We provide evidence that tumorigenesis in mice lacking p53 and p73 proceeds through mechanisms involving altered patterns of gene expression, defects in early T cell development, impaired apoptosis, and the ensuing accumulation of chromosomal aberrations. Collectively, our data imply that tumor suppressive properties of p73 are highly dependent on cellular context, wherein p73 plays a major role in T cell development and neoplasia.

## Introduction

The p73 gene was identified as a structural p53 homologue and a potential tumor suppressor [1], [2]. However, in contrast to the established role of p53 in cancer prevention, a similar role of p73 in oncogenesis remains to be explored. Despite the purported ability of p73 to activate p53-responsive genes, p73-deficient cells do not display a proliferative advantage similar to that of p53-null cells [3], [4]. The p73 gene is rarely mutated in human cancers, and no compelling evidence exists that p73 inactivation is required for tumor initiation or progression [5]. Apart from subsets of acute lymphoblastic leukemia and non-Hodgkin's lymphomas, few other human tumor types inactivate p73 through epigenetic silencing [6]–[8].

Unlike p53, the p73 gene locus gives rise to a complex pattern of isoforms with seemingly antagonistic properties. Many of the tumors upregulate the N-terminally truncated ΔNp73 isoforms, often in conjunction with transactivation competent full-length TAp73 [9]–[11]. Targeted inactivation of TAp73 in mice produces a phenotype similar to that of p53+/− mice in terms of incidence and latency of tumors [12]. In contrast, Trp73 knockout mice (which lack all p73 isoforms) exhibit multiple developmental defects but no tumor predisposition [13]. These data imply that TAp73 may act as a tumor suppressor, while ΔNp73 augments tumor development in vivo. In support, overexpression of ΔNp73 promotes cellular immortalization and oncogenic transformation, albeit with low efficiency compared to p53 dominant-negative mutants [14]. Also, upregulation of ΔNp73 is associated with poor prognosis in human cancers [5], [15].

An open question is whether p73 contributes to p53-mediated tumor suppression. One recent study showed that mice heterozygous for both p53 and p73 display higher tumor burden and metastasis compared to p53+/− mice [16], suggesting that mutations affecting p53 and p73 have additive effects in vivo. However, this gene interaction appears to be tissue-specific, as in a separate study p53+/− and p53+/−p73+/− mice exhibited no differences in radiation-induced lymphomagenesis [17]. Likewise, it was suggested that p73 loss impairs p53-dependent apoptosis in response to DNA damage [18]. However, p73 was found to be dispensable for p53-dependent apoptosis of T cells in vivo [3]. Moreover, the poor transforming properties of p73-deficient MEFs were seen as a consequence of compensatory activation rather than weakening of p53 [4]. Here, we set out to clarify this functional p53-p73 interrelationship by analyzing spontaneous tumorigenesis in mice deficient for both p53 and p73. Our data imply that the tumor suppressive properties of p73 are highly dependent on cellular context, wherein p73 plays a major p53-dependent role in T cell development and neoplasia.

## Results

### Mice Deficient in p53 and p73 Are Highly Prone to T Cell Lymphomas

**Figure 1:**
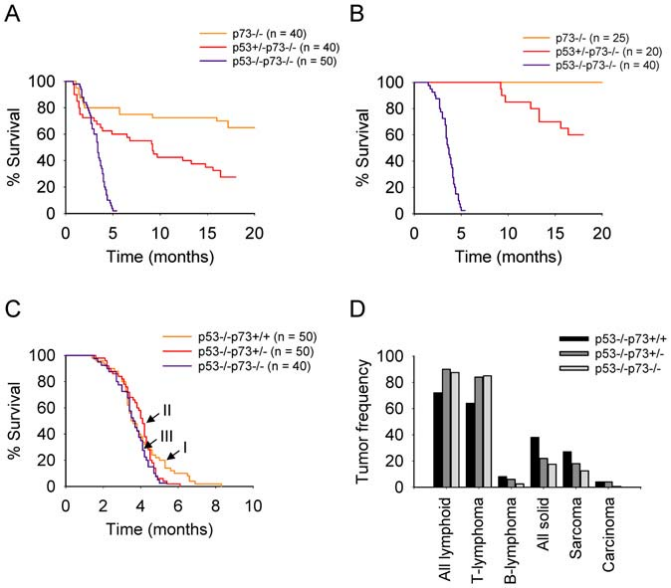
Mice deficient for p53 and p73 are highly prone to T cell lymphomas. **A, B.** Kaplan-Meier curves of overall (**A**) and tumor-free survival (**B**) of p73−/−, p53+/−p73+/− and p53−/−p73−/− mice. The number of mice of each genotype is indicated. **C.** Kaplan-Meier curves of tumor-free survival of p53−/−, p53−/−p73+/− and p53−/−p73−/− mice. **D.** Tumor spectra in mice of the indicated genotypes.

It was originally discovered and reported that p73-null mice survive to birth, but then exhibit a high rate of postnatal mortality caused by extensive brain defects, gastrointestinal hemorrhages, chronic infections and inflammation [13]. Therefore, animal studies to evaluate the long-term effect of p73 deficiency on cancer development were rendered not feasible. However, contrary to the accepted view, we found that growth retardation and the reduced postnatal survival of p73−/− mice were mainly due to malnutrition caused by their inability to compete with WT and p73+/− littermates. Identifying such p73−/− pups at an early stage and housing them with foster lactating mothers rescued nearly 80% of the mice to adulthood (>1.5 years, Fig. 1A). Thus far, we generated large cohorts of mice deficient for p73 and/or p53 and monitored them for developmental defects and tumor predisposition. In agreement with previous report [13], p73−/− mice in our group exhibited partially penetrant congenital hydrocephalus, and 20% of them died at an early age from intraventricular hemorrhage (Fig. 1A). Likewise, 25% to 30% of p53+/−p73−/− and p53−/−p73−/− (DKO) mice exhibited a shortened longevity due to inherent neurodevelopmental defects (Fig. 1A). Importantly, however, none of the long-term surviving p73−/− mice developed clinical signs of malignancy (Fig. 1B), in contrast to p53+/−p73−/− and DKO mice which succumbed to tumors at a later stage (Fig. 1B).

Mice lacking p53 are developmentally normal but tumor-prone, sustaining high rates of thymic lymphomas and other tumors [19], [20]. Most p53−/− mice in our group also succumbed to tumors within 2–6 months (Fig. 1C, curve I). Loss of one p73 allele had no significant effect on overall survival in this experimental setting (Fig. 1C, curve II), while loss of both p73 alleles shortened the lifespan of p53−/− mice, with DKO animals exhibiting a mean survival of 3.54+/−0.9 months compared to 3.92+/−1.4 months of p53−/− controls (Fig. 1C, curve III). Although the differences in terms of survival were small, loss of even one p73 allele significantly altered the tumor phenotype of p53-null mice. Thus, p53−/− mice developed thymic lymphomas (66%), high-grade sarcomas (26%), B cell lymphomas (8%), as well as occasional adenocarcinomas (4%), teratocarcinomas (4%), mesothelioma, and medulloblastoma (Fig. 1D and Table 1). In contrast, thymic lymphomas accounted for 82% and 83% of tumors found in p53−/−p73+/− and DKO mice, respectively (Fig. 1D and Table 1), whereas B cell lymphomas, sarcomas and carcinomas were less frequent in p53−/−p73+/− mice and even less so in DKO animals (Table 1). The differences in tumor frequencies between p53−/− and DKO mice were statistically significant (p<0.00091 and 0.00055 for T cell lymphomas and solid tumors, respectively, by Kendall's W test). Only five of forty (12.5%) DKO mice had various types of sarcomas, while adenocarcinomas were not found in DKO animals by the time they were 5 months old and other tumor types occurred (Fig. 1D and Table 1). Other, less frequent tumor types observed in DKO mice included low grade intestinal adenomas (5%) and malignant teratomas (7.5%), to which 129Sv mice have a modest predisposition [21].

**Table 1:** Tumor spectrum of p53−/− mice with zero, one, or two p73 alleles.

Genotypes			
Tumor types	p53−/− (n = 55)	p53−/−p73+/− (n = 57)	p53−/−p73−/− (n = 42)
T cell lymphoma	33 (66%)	42 (82%)	33 (83%)
B cell lymphoma	4 (8%)	3 (6%)	1 (2.5%)
NOS lymphoma^a^	3 (6%)	2 (4%)	1 (2.5%)
Angiosarcoma	11 (22%)	6 (12%)	4 (10%)
Fibrosarcoma	1 (2%)	1 (2%)	1 (2.5%)
Osteosarcoma	1 (2%)	–	–
Carcinosarcoma	–	1 (2%)	–
Stromal sarcoma	–	1 (2%)	–
Adenoma	–	1 (2%)	2 (5%)
Adenocarcinoma	2 (4%)	2 (4%)	–
Medulloblastoma	1 (2%)	–	–
Teratocarcinoma	2 (4%)	1 (2%)	3 (7.5%)
Inflammation	1	2	–
Cause not determined	4	4	2
Number of tumors	56	57	44
Tumors per mouse	1.12	1.12	1.1

The percentage of mice with tumors and the mean number of tumors per mouse are shown.

a NOS, not otherwise specified.

### Loss of p73 Enhances Dissemination of Lymphoma to Peripheral Organs

**Figure 2:**
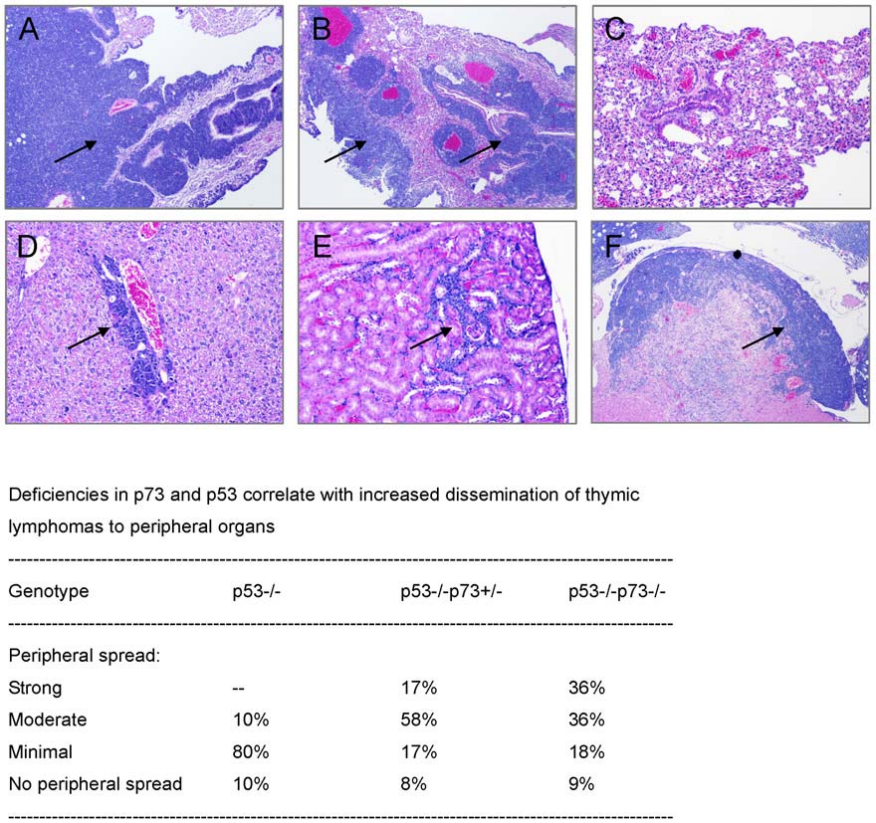
Loss of p73 enhances dissemination of lymphoma to peripheral organs. Hematoxylin and eosin staining of tissues from tumor-bearing p53−/−p73−/− mice showing lymphatic accumulation in lungs (**A, B**), liver (**D**), kidney (**E**), and heart (**F**). Lung section of a lymphoma-bearing p53−/− mouse is shown for comparison (**C**). Magnification 4X. The table lists the numbers of thymic lymphomas from mice with the indicated genotypes that either exhibited or lacked clear indications of invasion of peripheral organs by lymphoma.

A characteristic feature of lymphomas arising in p53−/− mice is that they remain within the thymus and only rarely spread to other organs (Fig. 2). By contrast, many of the lymphomas arising in p53−/−p73+/− mice and virtually all thymic tumors developed by DKO animals acquired the capacity to infiltrate adjacent structures and the periphery. Massive lymphatic accumulations were observed in mediastinal lymph nodes, lungs, and, on more rare occasions, in heart, liver, and kidneys of the affected animals (Fig. 2). At the cellular level, lymphomas arising in p53−/−p73+/− and DKO mice were invariably immature CD4+CD8+ double positive cells with a significant proportion of CD8-single positive cells, but few mature CD4-single positive cells. Most of these tumors expressed variable amounts of CD3, some of them showing a mixture of CD3-positive and CD3-negative T cells (data not shown). In sum, combined loss of p53 and p73 predisposes mice to lymphoblastic T cell lymphomas with widespread dissemination to peripheral organs.

### Loss of p73 Promotes T Cell Lymphomagenesis Independent of Age

**Figure 3:**
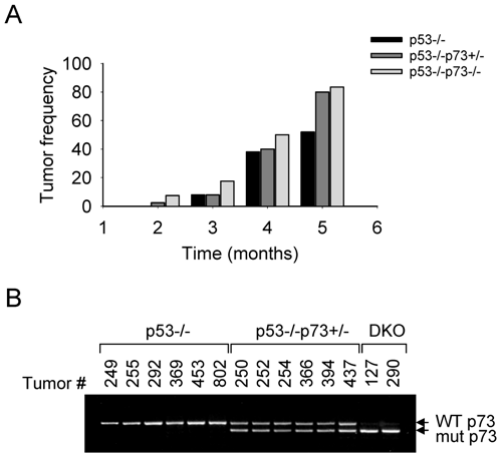
Loss of p73 promotes T cell lymphomagenesis independent of age. **A.** The dynamics of T cell lymphoma development in mice of the indicated genotypes. **B.** LOH analysis at the p73 locus by genomic PCR of tumor tissues derived from p53−/−, p53+/−p73−/−, and p53−/−p73−/− (DKO) mice. Tumor numbers are indicated.

We considered a possibility that the high frequency of lymphomas in DKO mice as compared to p53−/− mice may be due to a block in T cell development caused by the loss of p73 function. Alternatively, it could result from phenotypic variations in age-related thymic involution. However, thymi of 4- to 12-week-old p73−/− and DKO mice showed a histologically normal architecture, with well defined cortical and medullary areas (data not shown). Furthermore, atrophy of the thymus, as measured by total T cell reduction, was unaffected by the absence of p73 (Fig. S1A). In contrast, the onset of lymphoma development was accelerated in both p53−/−p73+/− and DKO mice (Fig. 3A). Importantly, no lymphomas arising in p53−/−p73+/− mice displayed LOH at the p73 locus (Fig. 3B), thus indicating that not only the complete loss of p73 but also a reduction of its dosage suffices to promote T cell lymphomagenesis independent of age.

Thymic involution in p53+/− mice occurs before appreciable numbers of thymocytes lose the remaining p53 allele [22]. Therefore, p53 heterozygous mice acquire tumors at older age, most often osteosarcomas and soft tissue sarcomas. As shown in Fig. 1B, the presence of one p53 allele alleviated the effects of p73 deficiency on tumor formation. However, while the control p53+/− mice succumbed mainly to high-grade sarcomas, thymic lymphomas were the major tumor type seen in p53+/−p73−/− mice (Fig. S1B, C).

### Inactivation of p73 Causes Defects in T Cell Development

T cell development is characterized by the progression through several phenotypically distinct stages, defined as double negative (DN), double positive (DP), and single positive (SP), based on the expression of CD4 and CD8 markers. The DN thymocyte subset is further subdivided into four stages (DN1-4) by differential expression of CD25 and CD44 [23]. To determine whether p73 loss affects T cell development, lymphoid organs (bone marrow, thymi, spleens) of p73−/− and DKO mice were characterized by flow cytometry and PCR.

**Figure 4:**
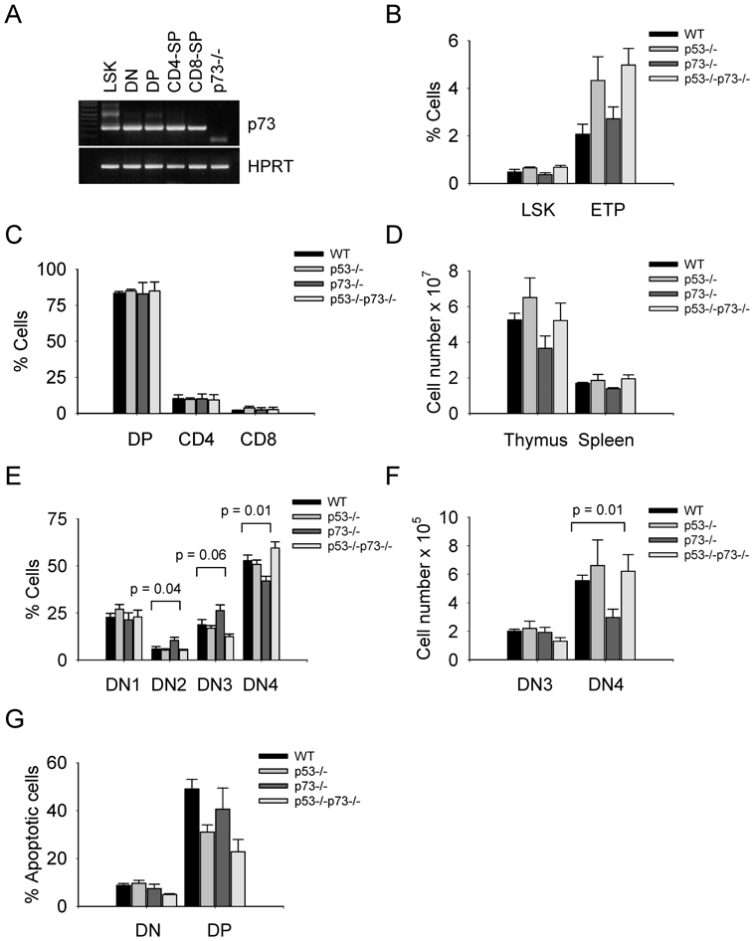
Inactivation of p73 causes defects in T cell development. **A.** Semi-quantitative RT-PCR analysis of p73 mRNA levels in sorted bone marrow Lin-Sca1+c-Kit+ (LSK) cells, thymic CD4/CD8 double negative (DN), CD4/CD8 double positive (DP), CD4-SP and CD8-SP cells from WT mice. HPRT is a control for equal input. p73−/− thymocytes are shown for control. **B.** The frequency of LSK lymphoid progenitors in bone marrow and early T lineage progenitors (ETP) in thymi of mice of the indicated genotypes. ETP cells represent a c-Kit-positive subset of the DN1 (CD44+CD25-) population. The error bars represent the standard error obtained from six experiments. **C.** Relative numbers of DP, CD4-SP and CD8-SP T cell in thymi from 4-week old mice of the indicated genotypes. The error bars represent the standard error. **D.** Total cell numbers in thymi and spleens of 4 week-old mice of the indicated genotypes. The error bars represent the standard error obtained from six experiments. **E, F.** Thymocytes from 4-week old mice of the indicated genotypes were stained with antibodies to CD4, CD8, CD25 and CD44. Gating on CD4/CD8 double negative cells. Relative numbers of DN1-4 (**E**) and absolute numbers of DN3/DN4 subsets (**F**) are indicated. The error bars represent the standard error obtained from eight experiments. P-values for the difference between WT and p73−/− subsets are shown. **G.** Annexin V staining of DN and DP thymocytes from 4 week-old mice of the indicated genotypes. The error bars represent the standard error obtained from three experiments.

The analysis revealed that p73 is expressed throughout T cell development (Fig. 4A). The frequency of primitive lymphoid progenitors (LSK) in bone marrow and early T lineage progenitors (ETP) in thymi of p73−/− and DKO mice was normal (Fig. 4B). The proportions of DP, CD4-SP and CD8-SP thymic subsets were also comparable with those of controls (Fig. 4C). However, the total number of thymocytes in p73−/− mice was only 70% that of wild-type littermates (Fig. 4D). Deletion of p73 caused a partial arrest at the DN2/DN3 stage and reduced the numbers of DN4 pro-T cells (Fig. 4E, F). DN2 and DN3 thymocytes that escaped this partial developmental arrest retained the ability to generate DN4 cells and undergo further differentiation (Fig. 4C). However, in isolated cases spleens of p73−/− mice were devoid of T cells (data not shown). In view that the generation of double strand breaks during V(D)J recombination poses a risk for genomic instability in DN2/DN3 and DP stage thymocytes and accumulation of mutations [24], we surmised that the DNA damage response and apoptosis might be disrupted upon combined inactivation of p53 and p73. As reported [3], thymocytes of p73−/− mice displayed only slightly reduced levels of apoptosis compared to WT controls, but it was lessened by the deletion of p53 (Fig. 4G). The developmental arrest imposed by p73 deficiency was also rescued by the deletion of p53 (Fig. 4E, F), restoring thymic cellularity to WT control levels (Fig. 4D).

**Figure 5:**
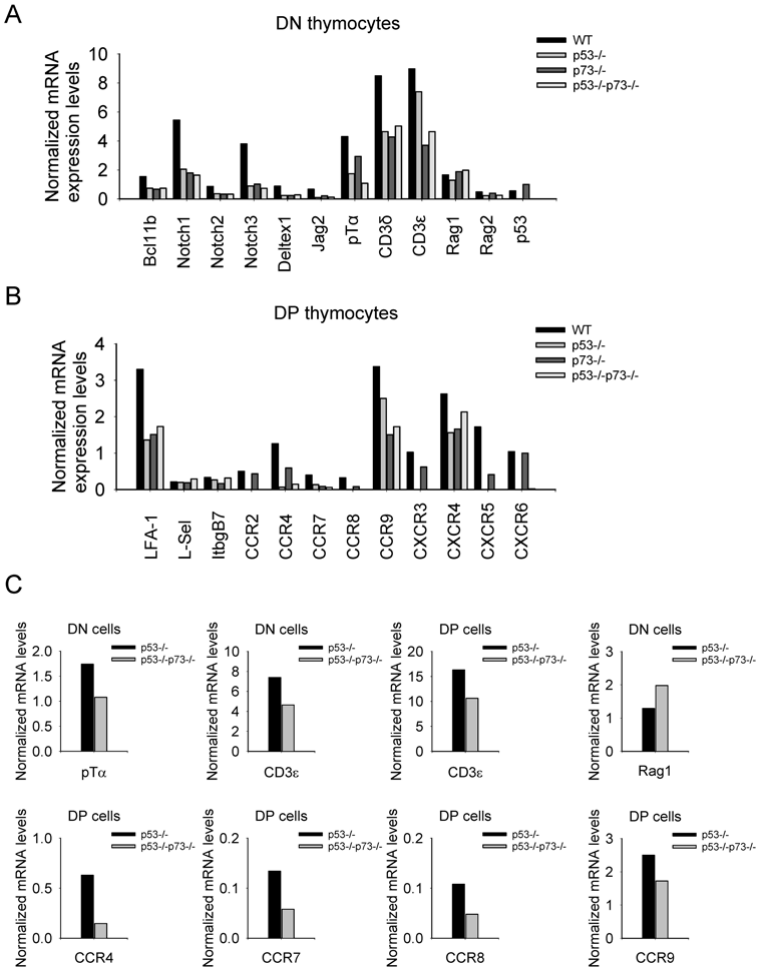
Loss of p73 alters the expression pattern of genes involved in T cell development. **A–C.** Quantitative RT-PCR analysis of genes encoding critical factors of T cell development in DN and DP thymocytes of the indicated genotypes. Each sample was analyzed in duplicate. mRNA expression levels were normalized to hypoxanthine-guanine phosphoribosyl transferase (HPRT) mRNA amount.

To explore whether the early T cell developmental program takes place correctly in thymocytes of p73-deficient mice, the expression of the genes involved in early cell-fate decisions and differentiation processes was examined by quantitative RT-PCR. In these experiments, mRNA levels of Bcl11b, Notch1, Notch3 and pre-TCR components (pTα, CD3δ, CD3ε) in DN thymocytes of p73−/− mice were markedly reduced compared with those of WT control mice (Fig. 5A), and this expression pattern was maintained until the DP stage (Fig. S2A-C). In contrast, p53 mRNA levels were increased twofold in p73−/− DN cells (Fig. 5A). Likewise, p73-deficient thymocytes contained altered mRNA levels of LFA-1 (integrin beta 2) and chemokine receptors CCR4, CCR7, CCR8, CCR9 and CXCR5 (Fig. 5B), which have been implicated in lymphocyte migration and dissemination [25]. Expression levels of pTα, CD3ε, CCR4, CCR7, CCR8, CCR9 and CXCR3 genes were also lower in DKO compared to p53−/− cells (Fig. 5B, C and Fig. S2A-C). Similarities between p53−/− and DKO DP thymocytes included conspicuously low mRNA levels of Notch1-3 and chemokine receptors CCR2, CCR4, CCR7, CCR8, CXCR3, CXCR5, and CXCR6 (Fig. 5B). Thus, loss of p73 synergizes with p53 loss-of-function in altering the developmental and/or migratory properties of immature T cells.

### Aneuploidy and Clonal Translocations Contribute to the Development of p73-Deficient Lymphomas

**Figure 6:**
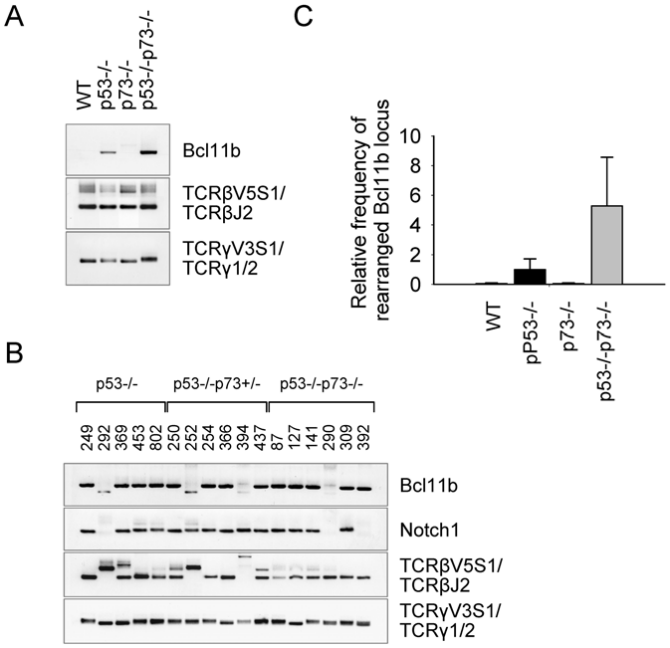
V(D)J recombination poses a risk for genomic instability in thymocytes deficient for p53 and p73. **A, B.** Genomyc PCR analysis of internal deletion at the Bcl11b and Notch1 loci in thymocytes from WT, p53−/−, p73−/−, and DKO mice (**A**), and in lymphomas derived from p53−/−, p53−/−p73+/− and p53−/−p73−/− mice (**B**). Consistent data were obtained from three mice of each genotype. TCRβ and TCRγ gene rearrangements are shown for controls. Tumor numbers are indicated. **C.** Relative frequency of rearranged Bcl11b gene in thymocytes from WT, p53−/−, p73−/−, and DKO mice. The results are representative of six mice of each genotype. The error bars represent the standard error.

At the genomic level, rearrangements involving TCRβ and TCRγ gene loci were detected in premalignant thymocytes of all genotypes (Fig. 6A), and in lymphomas derived from p53−/−, p53−/−p73+/− and DKO mice (Fig. 6B). In contrast, interchromosomal translocations between TCRγ and TCRβ loci that have been used routinely as predictors for large-scale translocations in mouse thymocytes [26], [27], were not present in cells of either genotype (data not shown). Evidence for aberrant V(D)J rearrangements in 73-deficient thymocytes was obtained by assaying for internal deletion at the Bcl11b and Notch1 loci, which both contain cryptic recombination signal sequences (RSS) that are accessible to Rag, and are frequently mutated in T cell lymphomas [28]–[30]. Deletions at the Bcl11b gene locus were present in premalignant p53−/− and DKO thymocytes (Fig. 6A) and in lymphomas derived from p53−/−, p53−/−p73+/− and DKO mice (Fig. 6B). On the other hand, deletions at the Notch1 locus were found in the respective lymphomas but not in premalignant cells (Fig. 6A, B). The frequency of Bcl11b gene rearrangements in DKO mice was estimated to be 2 to 5-fold higher than that in the respective p53−/− controls (Fig. 6C). PCR of serial dilutions showed that rearrangement of the Bcl11b gene could be reliably detected in 300 ng of thymocyte DNA derived from premalignant p53−/− mice and 150 ng DNA from DKO mice (data not shown). Considering that this amount of thymocyte DNA corresponds to ∼5×10^4^ and 2.5×10^4^ cell equivalents, respectively (Sakata et al., 2004), and the normal thymus is composed of ∼5×10^7^ cells (see Fig. 4D), our results suggest the existence of at least 10^3^ thymocytes with internal deletions in the Bcl11b gene in thymi of premalignant p53−/− mice and twice that number in thymi of DKO mice.

To examine chromosomal alterations associated with p53 and p73 deficiency in more detail, array-based comparative genomic hybridization (CGH) assays were performed on five primary tumors of each p73 genotype. The analysis revealed that p53−/−p73+/− and DKO lymphomas resembled p53-null tumors in terms of extensive numerical chromosomal aberrations. Whole chromosome gains of 4, 9, 11, 14, and 15 were common in all types of lymphomas, indicating that tumor development in a p53-null background favors the duplication of these chromosomes (Fig. S3). Distinct karyotypic events were also evident, as gains of chromosome 1 were more frequent in p73-deficient lymphomas (Fig. S3). Spectral karyotyping (SKY) of cell lines established from p53−/−p73+/− and DKO lymphomas revealed a modal chromosome number of 46-52 accompanied by several types of translocations, including t(8; 11), t(1; 10), t(13; 16), and t(16;17) (Fig. S4). Based on these data, we conclude that tumorigenesis in p53−/−p73+/− and DKO mice proceeds through mechanisms involving chromosome gains, losses, and clonal translocations. The contribution of numerical chromosomal changes is more than that of chromosomal translocations.

## Discussion

We show here that p73 acts as a T cell-specific tumor suppressor in a genetically defined mouse model, and that concomitant ablation of p53 and p73 predisposes mice to an increased incidence of thymic tumors compared to the loss of p53 alone. Our results demonstrate a causal role for loss of p73 in progression of T cell lymphomas to the stage of aggressive, disseminated disease. We provide evidence that tumorigenesis in mice lacking p53 and p73 proceeds through mechanisms involving altered patterns of gene expression, defects in early T cell development at the immature DN2-DN4 stage, impaired apoptosis, and the ensuing accumulation of chromosomal aberrations, i.e. chromosome gains, losses, and clonal translocations. We further demonstrate that p73 loss alone cannot initiate tumorigenesis but it can cooperate with an initiating lesion, such as p53 loss-of-function, to make a premalignant cell tumorigenic and proliferatively aggressive. Based on the fact that B cell lymphomas, carcinomas and sarcomas occurred infrequently in p53−/−p73+/− mice and even less so in p53−/−p73−/− (DKO) animals, our data imply that tumor suppressive properties of p73 are highly dependent on cellular context, wherein p73 plays a major p53-dependent role in T cell development and neoplasia.

There are two current concepts of the cellular mechanisms responsible for the induction and maintenance of T cell lymphomas. One holds that p53 participates in a checkpoint response that eliminates DN thymocytes that do not display proper pre-TCR signaling. Cells that fail to express the pre-TCR exhibit developmental arrest at the DN3 stage, accompanied by massive apoptosis [31]–[34]. Ablation of p53 function can restore the survival of pre-TCR-deficient thymocytes, but at later time points these cells consistently give rise to lymphomas [32], [34], [35]. Another model holds that any genetic alteration leading to an early lymphocyte block and prolonged expression of Rag endonucleases can act as a procarcinogenic event, particularly in the p53 null setting [36], [37]. We present several lines of evidence to support a model in which disease acceleration as a direct or indirect consequence of p73 inactivation is caused by the alteration of expression patterns of genes involved in early cell-fate decisions, most notably pTα, CD3ε, Bcl11b, and Notch1. Accumulating evidence has highlighted the importance of these genes in the regulation of differentiation of lymphoid progenitor cells, the pre-TCR checkpoint and T cell-specific gene expression [28], [38], [39]. As previously reported [3], p73−/− mice produce a normal repertoire of mature T cells. However, we find that from a mechanistic perspective p73 deficiency perturbs early T cell development and thereby increases the number of T cell progenitors available for secondary mutations. While only a small percentage of such cells progress beyond the DN stage due to persistent p53-mediated cell cycle arrest and apoptosis, the deletion of p53 rescues this defect, but it comes at the expense of increased lymphomagenesis. Furthermore, p73-deficient thymocytes exhibit altered mRNA levels of chemokine receptors such as CCR4, CCR7, CCR8, CCR9, and CXCR3, which have been implicated in governing lymphocyte migration and dissemination [25].

Our findings implicate Rag activity as an important contributing factor affecting lymphoma development in a p73-null background, while the loss of apoptosis through p53- and p73-deficiency allows accumulation of such genetic alterations. First, thymopoiesis in p73−/− mice is blocked at the DN3 stage that normally expresses Rag. Second, Rag-mediated internal deletions in the Bcl11b gene locus were detected in premalignant DKO thymocytes. We find that the frequency of Bcl11b gene deletions is twofold higher in premalignant DKO cells as compared with p53-null controls. Third, Rag-mediated internal deletions in the Notch1 locus were detected in DKO lymphomas, suggesting continuous Rag gene activity during the evolution of the transformed clones. In sum, although p53−/− and DKO lymphomas did not harbor Rag-mediated translocations of the TCR loci, our findings support a model in which persistent Rag expression contributes to tumorigenesis in an overall growth arrest-deficient and apoptosis-deficient p53−/−p73−/− null background. This model is consistent with previous reports that linked lymphomagenesis with Rag-mediated genomic alterations [28], [36], [37].

The increased propensity to dissemination is a salient feature of p73-null lymphomas. We showed recently that p73 loss alters the clinical course of Myc-driven lymphomagenesis, characterized by a decreased tumor burden in lymphoid organs, but increased dissemination to extranodal sites (A Nemajerova and U Moll, submitted). Microarray analysis of p73-deficient lymphomas revealed a number of deregulated genes that encode proteins involved in cell adhesion and extracellular signaling. These data provide strong evidence that p73 loss alters gene expression patterns of various lineages. Because transcriptional silencing of the p73 gene through methylation has been demonstrated in human leukemias and lymphomas [6]–[8], it will be important to establish if p73 plays a similar role in regulation of T cell development and neoplasias in humans.

## Materials and Methods

### Ethics Statement

All research involving animals has been conducted according to national and international guidelines with respect to husbandry, experimentation and welfare as part of this project. All animal studies were approved by the Institutional Animal Care and Use Committee at Stony Brook University.

### Mice and Flow Cytometry

We used previously described p73-knockout mice [13] and p53-knockout mice [20]. All mice were housed in the maximum isolation facility. p53+/−p73+/− double heterozygous mice were derived by crossing between p73+/− and p53−/− animals, both on the 129S1/SvImJ background. p53−/−p73+/− mice were derived from crosses between the double heterozygous animals. p53−/−p73−/− mice (DKO) were derived from crosses between p53+/−p73+/− double heterozygous mice or p53+/−p73+/− and p53−/−p73+/− mice. The genotype of mice was verified by PCR amplification specific for the corresponding WT and mutant alleles. Mice were observed daily for precipitous changes in health and tumor development. Tumors that arose were harvested and processed for histological examination. Each tumor was fixed in formalin and sections were stained with hematoxylin and eosin. The extent of tumor dissemination was determined for at least 15 lymphomas of each genotype and graded the percentage of lung tissue invaded by lymphoma cells per 10X power field. Dissemination was classified into 4 grades: 0 = no signs of dissemination, 1 = minimal (<5% invaded tissue), 2 = moderate (up to 20% invaded tissue), 3 = strong (>30% invaded tissue). Lymphoid tissue samples were examined by flow cytometry with anti-CD3 (145-2C11), CD4 (H129.19 and GK1.5), CD8 (53–67), CD19 (MB19-1 and 1D3), CD25 (7D4), CD44 (IM7), B220 (CD45R) (RA3-6B2), TCRβ (H57-597), TCRγ (UC7-13D5), c-Kit (ACK2 and 2B8), and Sca1 (D7) antibodies (BD Pharmingen and eBiosacince). Kendall's coefficient of concordance, Wilcoxon matched pairs test, and Student's t-test were used in the statistical evaluation. For cell-cycle analysis, lymphoid cells were fixed in 70% ethanol, stained with propidium iodide and analyzed using FACS Calibur (Becton-Dickinson) with CellQuest software. Annexin V staining was performed as recommended by the manufacture (Roche).

### DNA and Expression Analyses

Comparative genomic hybridization (CGH) and Spectral karyotyping (SKY) were performed at the Roswell Park Cancer Institute following published procedures [40], [41]. For SKY analysis, metaphase chromosomes were prepared from tumor cell lines at early-passage numbers. Ten to twenty metaphases were analyzed for each tumor. To assay for recombinations within and between TCRG and TCRB loci, nested PCR was performed as described previously [26]. Deletions between breakpoint regions at positions 4926 and 16674 of the mouse Notch1 gene were examined as described [29]. To assay for Bcl11b internal deletions, nested PCR was performed using primers homologous to the major breakpoint regions within introns 1 and 3 [28]. The identities of amplified PCR fragments were confirmed by DNA sequencing. Semiquantitative and Real-Time RT-PCR analyses were performed using whole cell RNAs prepared from primary lymphoid cells or tumors and previously described oligonucleotide primers. The sequences of primers are listed in Table S1. Relative expression of the target genes was calculated using the ΔCT method described previously: Relative expression = 2^−ΔCT^, where ΔCT = CT (Target gene) - CT (HPRT).

## Supporting Information

Figure S1 A. Total cell numbers in thymi of ageing mice of the indicated genotypes. The error bars represent the standard error obtained from three experiments. B. Kaplan-Meier curves of tumor-free survival of p53+/− and p53+/−p73−/− mice. The number of mice of each genotype is indicated. C. Tumor spectra in mice of the indicated genotypes.
(0.57 MB TIF)

Figure S2 Quantitative RT-PCR analysis of genes encoding critical factors of T cell development in DN (A) or DP thymocytes (B, C) of the indicated genotypes. Each sample was analyzed in duplicate. mRNA expression levels were normalized to hypoxanthine-guanine phosphoribosyl transferase (HPRT) mRNA amount.
(0.06 MB PDF)

Figure S3 CGH analysis of chromosomal alterations in primary T cell lymphomas from p53−/−, p53-p73+/− and p53−/−p73−/− mice. The results are representative of five tumors of each genotype. Chromosomal gains are shown in green, while chromosomal losses are shown in red. The blue boxes indicate the most common chromosomal aberrations.
(1.25 MB TIF)

Figure S4 SKY analysis of cell lines established from p53−/−, p53-p73+/− and p53−/−p73−/− T cell lymphomas. Chromosomes with numerical changes (white circles) and translocations (yellow circles) are indicated.
(2.99 MB TIF)

Table S1 Primers for quantitative RT-PCR.
(0.04 MB DOC)
