# Supplementary figures and images for: Targeted Deletion of p73 in Mice Reveals Its Role in T Cell Development and Lymphomagenesis
Source: PLoS One. 2009 Nov 11;4(11):e7784. doi: 10.1371/journal.pone.0007784 (PMC2771421; doi:10.1371/journal.pone.0007784)

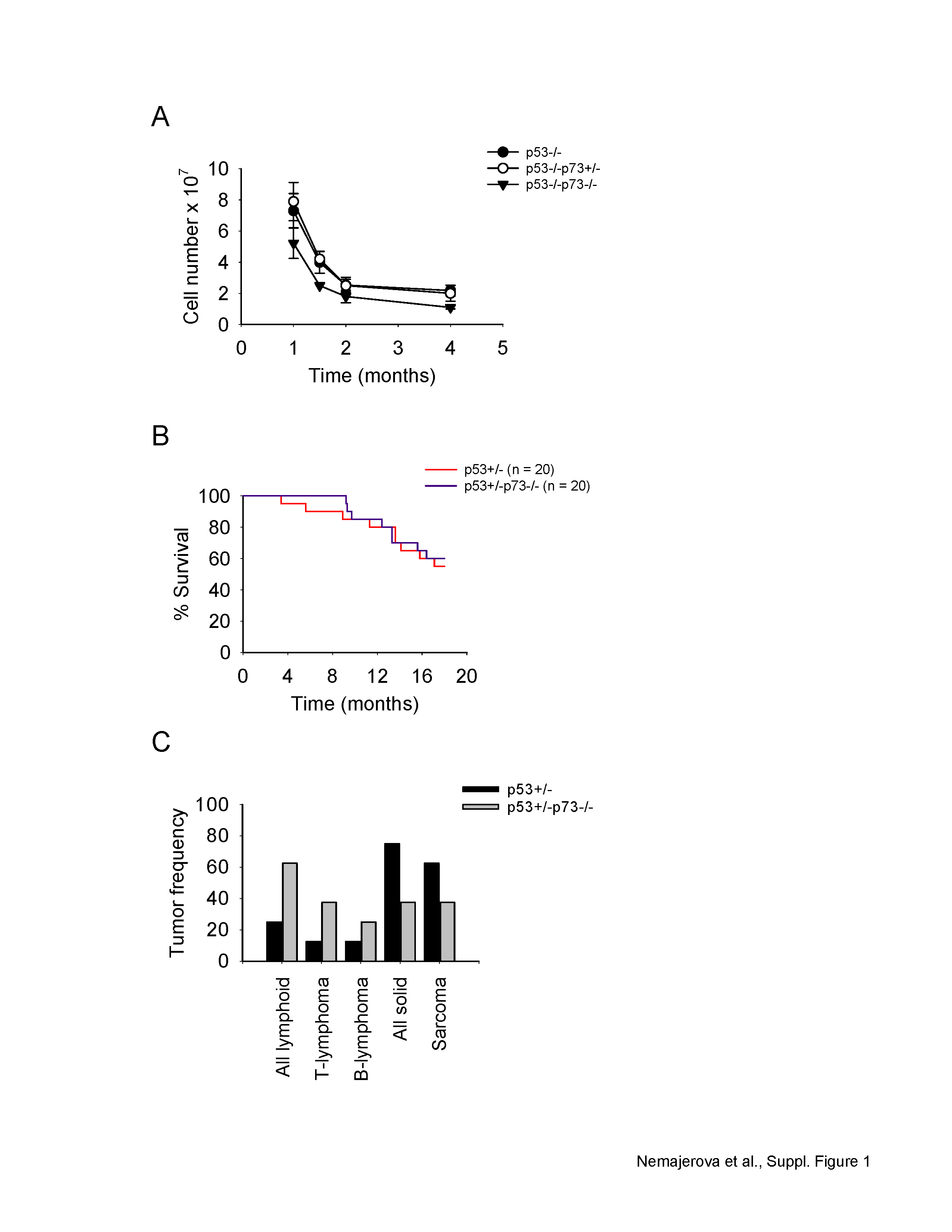

Supplement: Figure S1 — A. Total cell numbers in thymi of ageing mice of the indicated genotypes. The error bars represent the standard error obtained from three experiments. B. Kaplan-Meier curves of tumor-free survival of p53+/− and p53+/−p73−/− mice. The number of mice of each genotype is indicated. C. Tumor spectra in mice of the indicated genotypes. (0.57 MB TIF) [file pone.0007784.s001.tif]

**A**

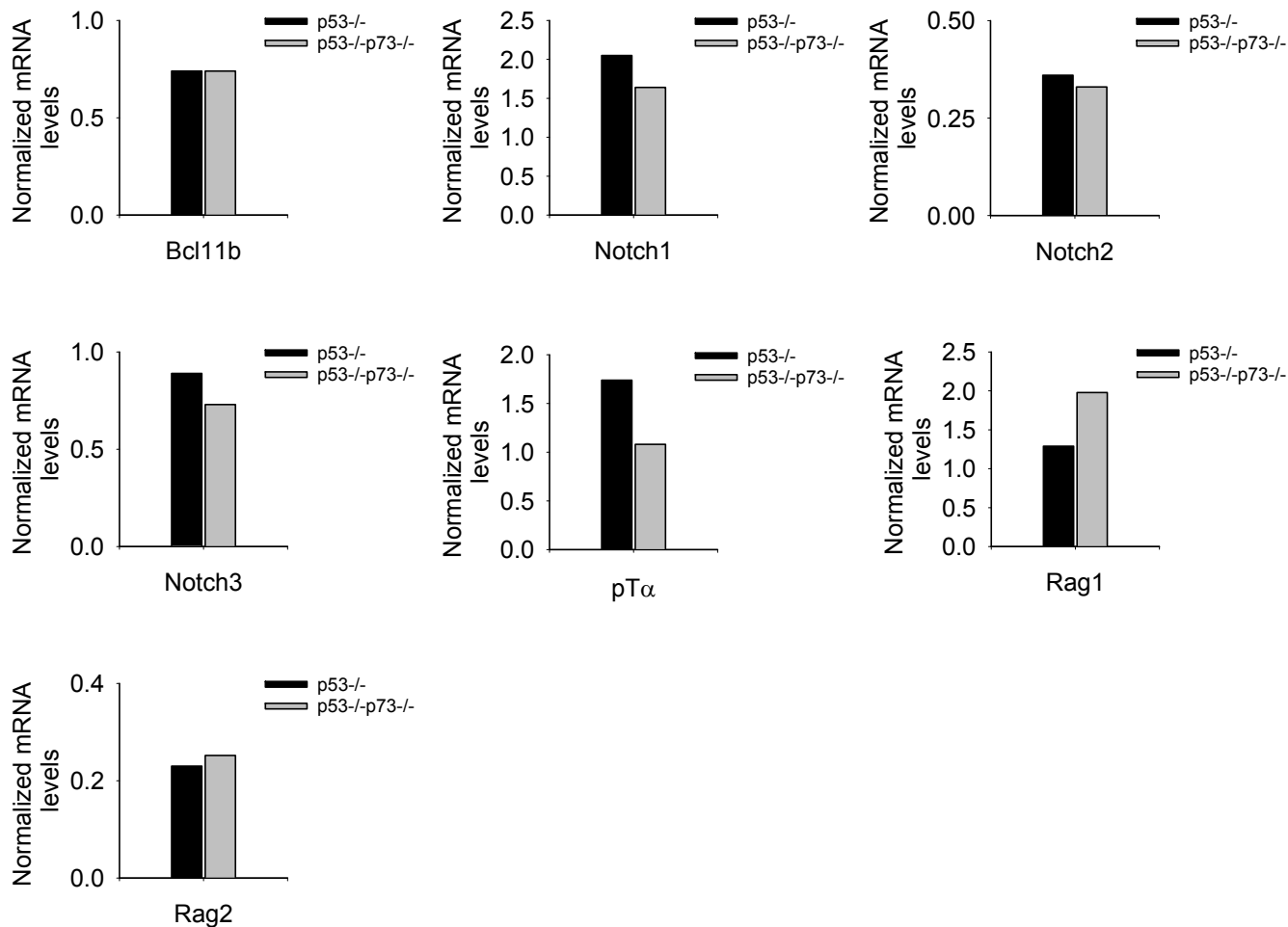

**B**

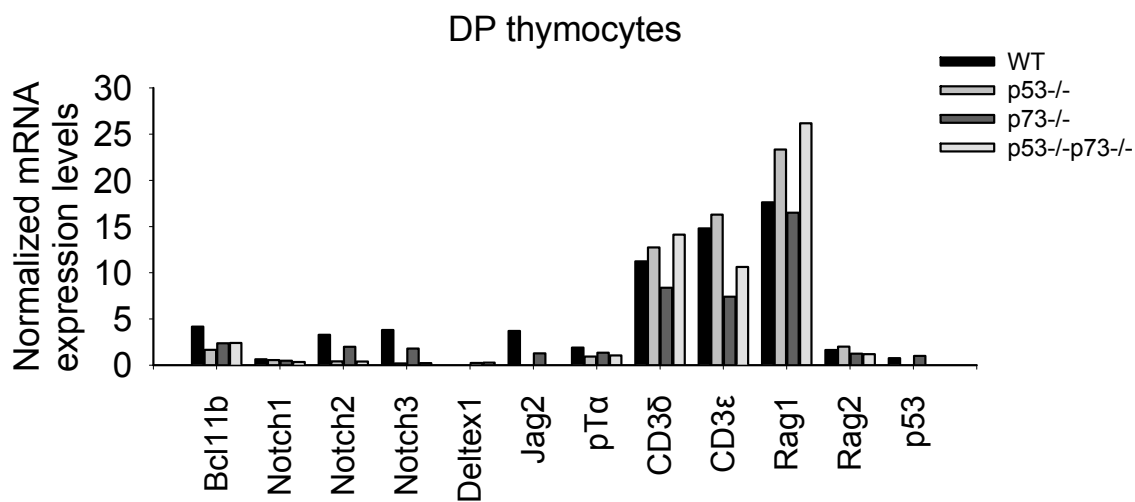

C

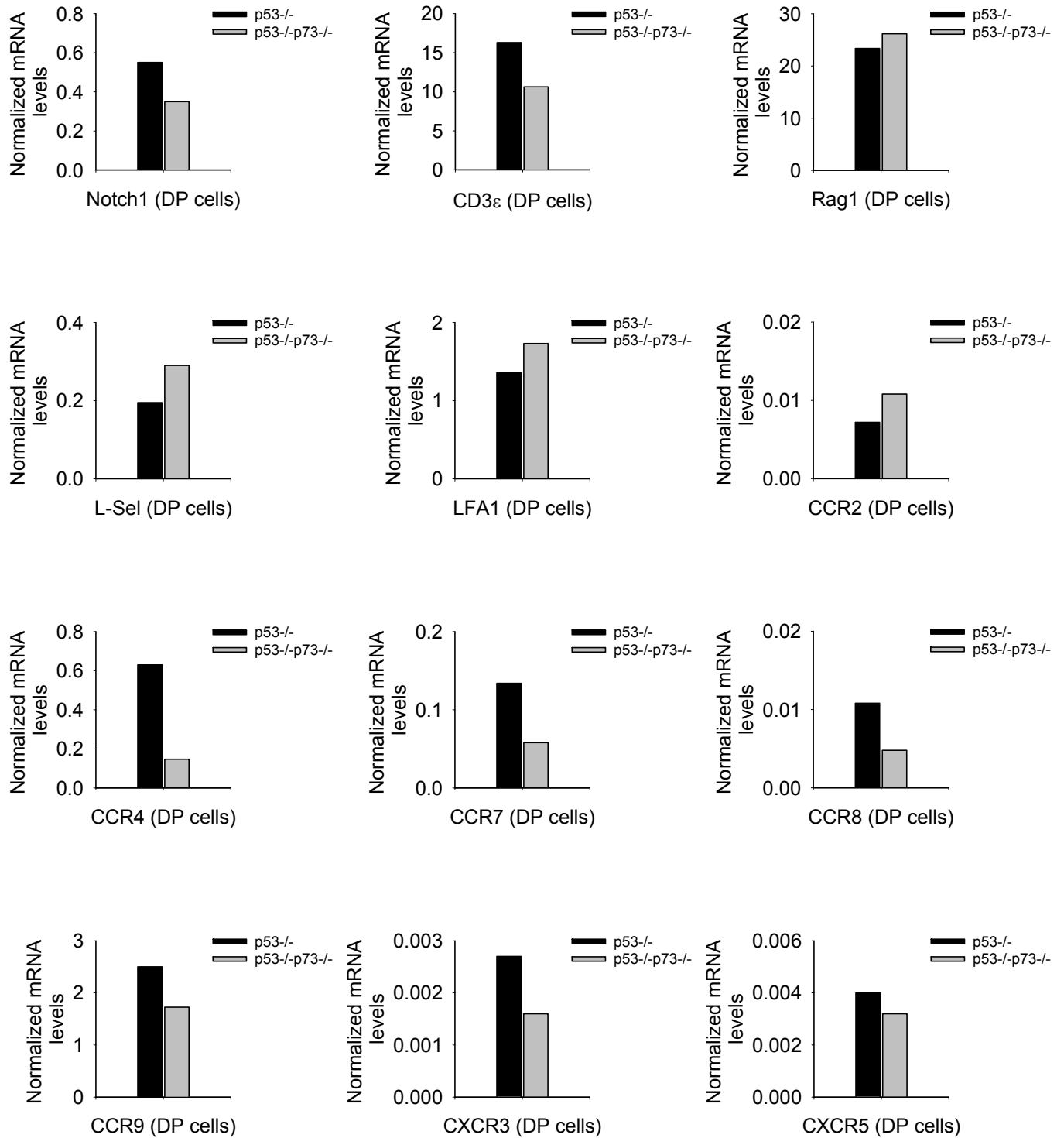

Supplement: Figure S2 — Quantitative RT-PCR analysis of genes encoding critical factors of T cell development in DN (A) or DP thymocytes (B, C) of the indicated genotypes. Each sample was analyzed in duplicate. mRNA expression levels were normalized to hypoxanthine-guanine phosphoribosyl transferase (HPRT) mRNA amount. (0.06 MB PDF) [file pone.0007784.s002.pdf]

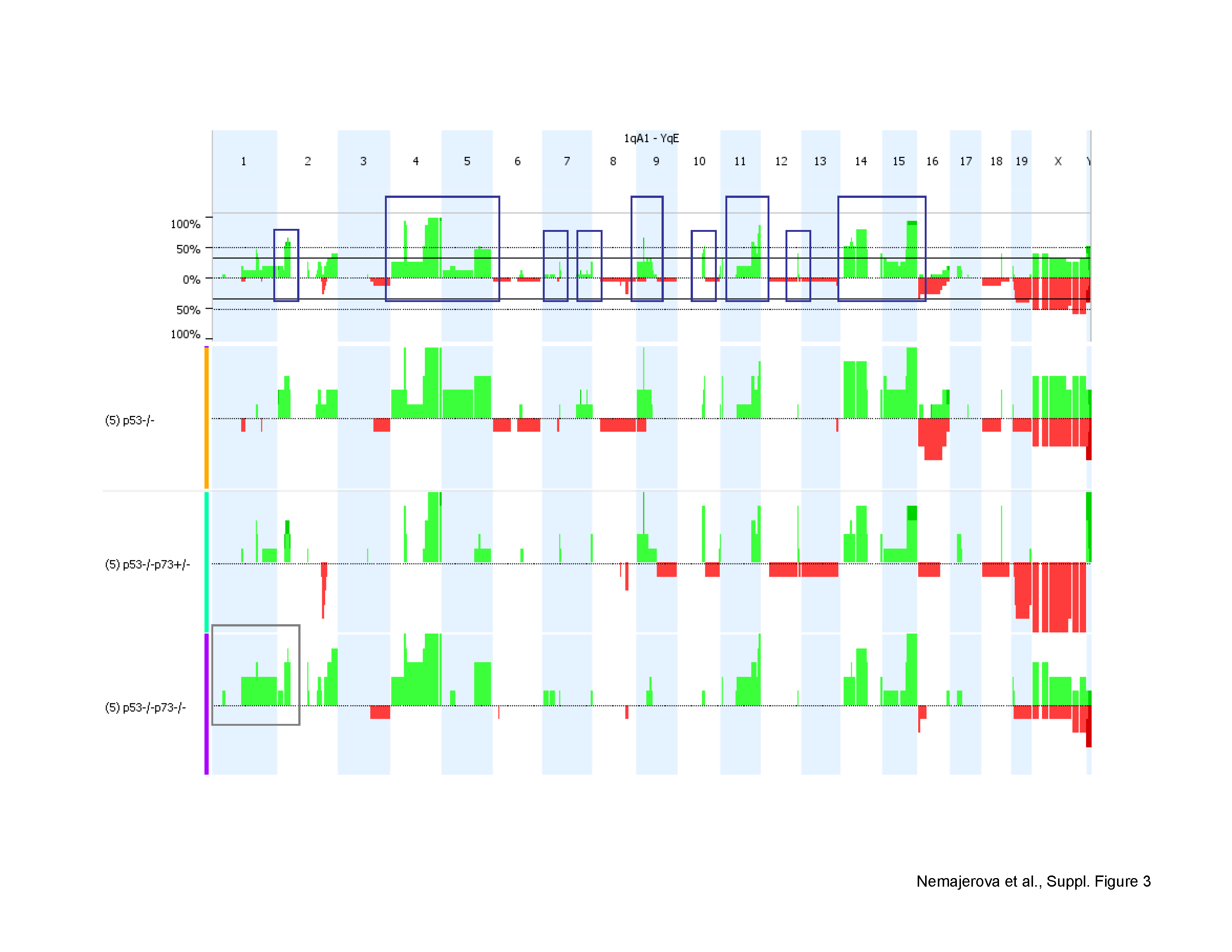

Supplement: Figure S3 — CGH analysis of chromosomal alterations in primary T cell lymphomas from p53−/−, p53-p73+/− and p53−/−p73−/− mice. The results are representative of five tumors of each genotype. Chromosomal gains are shown in green, while chromosomal losses are shown in red. The blue boxes indicate the most common chromosomal aberrations. (1.25 MB TIF) [file pone.0007784.s003.tif]

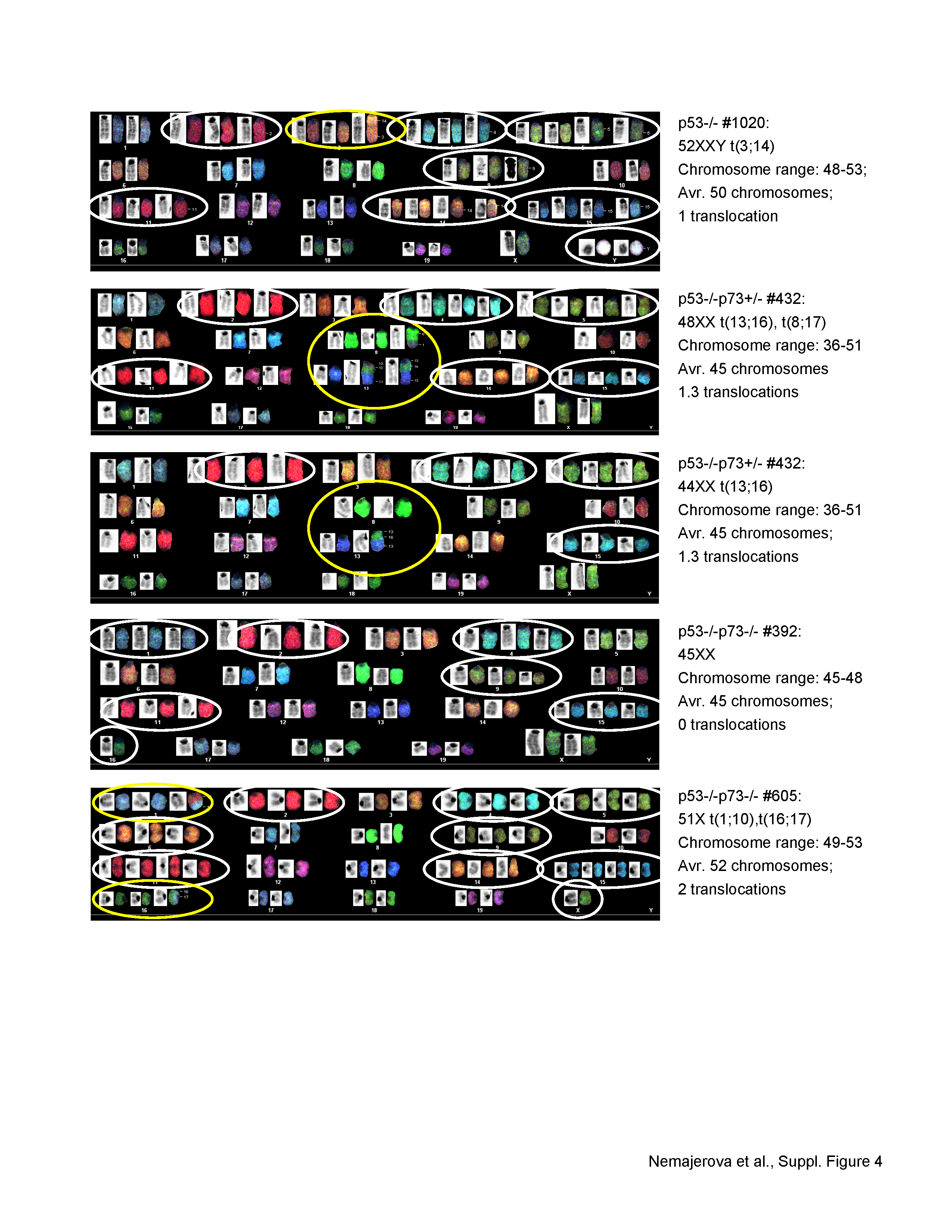

Supplement: Figure S4 — SKY analysis of cell lines established from p53−/−, p53-p73+/− and p53−/−p73−/− T cell lymphomas. Chromosomes with numerical changes (white circles) and translocations (yellow circles) are indicated. (2.99 MB TIF) [file pone.0007784.s004.tif]
