# Supplementary material for: Targeted Deletion of p73 in Mice Reveals Its Role in T Cell Development and Lymphomagenesis
Source: PLoS One. 2009 Nov 11;4(11):e7784. doi: 10.1371/journal.pone.0007784 (PMC2771421; doi:10.1371/journal.pone.0007784)
Supplement: Table S1 — Primers for quantitative RT-PCR. (0.04 MB DOC) [file pone.0007784.s005.doc]

**Supplemental Table 1.**

Primers for quantitative RT-PCR.

CCR2

5′-AACAGTGCCCAGTTTTCTATAGG-3′

5′-CGAGACCTCTTGCTCCCCA-3′

CCR4

5′-ATCCTGAAGGACTTCAAGCTCCA-3'

5′-AGGTCTGTGCAAGATCGTTTCATGG-3'

CCR5

5′-CTCCCAGAAATAATCTTTAC-3’

5′-TCAGGCTCAAGATGACCATC-3’

CCR6

5′-CCTCACATTCTTAGGACTGGAGC-3’

5′-GGCAATCAGAGCTCTCGGA-3’

CCR7

5′-AAAGCACAGCCTTCCTGTGT-3’

5′-AGTCCACCGTGGTATTCTCG-3’

CCR8

5′-CAGATAATTGGTCTTCCTGCCTC-3’

5′-TGAGGAGGAACTCTGCGTCACA-3’

CCR9/10

5′-CAGCCTTATTCCTGGCATGT-3’

5′-CTTGCAAACTGCCTGACATT-3’

CXCR3

5′-TGCTAGATGCCTCGGACTTT-3'

5′-CGCTGACTCAGTAGCACAG-3′

CXCR4

5′-TCAGTGGCTGACCTCCTCTT-3′

5′-CTTGGCCTTTGACTGTTGGT-3’

CXCR5

5'-ACTCCTTACCACAGTGCACCTT-3'

5'-GGAAACGGGAGGTGAACCA-3'

CXCR6

5′-ATGTTTGCCCCAACAGATG-3'

5'-CTACAATTGGAACATACTGGTGG-3'

L-selectin-c

5’-CATTCCTGTAGCCGTCATGGT-3’

5’-TCTTGAGATTTCTTGCCTTTTTTTAAC-3’

LFA-1 (Itgb2)

5′-CCGACAACTCCAACCAGTTT-3’

5′-AGCAGCCTCGTGACATTGCGC-3’

Integrin b7 (Itgb7)

5′-CAGCCACCCTTCAGCTTTCACC-3’

5′-CCAGCCAATCTGTTCCTGGCAG-3’

pTα

5'-ACCATCAGGCATCGCTGGC-3'

5'-CGAGGACCAGGCAAACCACC-3'

CD3ε

5'-TCTCGGAAGTCGAGGACAGT-3'

5'-GCTCATAGTCTGGGTTGGGA-3'

CD3δ

5'-AGCGGGATTCTGGCTAGTCT-3'

5'-GTCTCATGTCCTGCAAAGCA-3'

TdT

5'-AGAGACCTTCGGCGCTATG-3'

5'-TGACAGTCTTCCCCTTAGTCC-3'

Rag1

5'-ACCCGATGAAATTCAACACCC-3';

5'-CTGGAACTACTGGAGACTGTTCT-3';

Rag2

5'-AAGGCTGGCCTAAGAGATCCT-3';

5'-GATAACGAAGAGGTGGGAGGTA-3';

Nrarp

5'- AAGCTGTTGGTCAAGTTCGGA-3'

5'- CGCACACCGAGGTAGTTGG-3'

Deltex

5'- ATCAGTTCCGGCAAGACACAG-3'

5'- CGATGAGAGGTCGAGCCAC-3'

Bcl11b

5'-CAGATCGGCAAGGAGGTGTA-3'

5'-AGGACTTCGCAGACACAGGTTA-3'

Notch1

5'-CCCTTGCTCTGCCTAACGC-3';

5'-GGAGTCCTGGCATCGTTGG-3';

Notch2

5'-ATGTGGACGAGTGTCTGTTGC-3';

5'-GGAAGCATAGGCACAGTCATC-3';

Notch3

5'-TGCCAGAGTTCAGTGGTGG-3';

5'-CACAGGCAAATCGGCCATC-3';

Jag1

5'-CAAAGTGTGCCTCAAGGAGTATCAG-3'

5'-TCCACCAGCAAAGTGTAGGACCTC-3'

Jag2

5'-CAAGTTGTGTGACGAGTGTGTCCC-3'

5'-TTGCCCAAGTAGCCATCTGG-3'

c-Myc

5'- GTCCTGGATGATGATGTTCT-3'

5'- AGGAAGAGAATTTCTATCAC-3'

HPRT

5'-GGGGGCTATAAGTTCTTTGC-3'

5'-TCCAACACTTCGAGAGGTCC-3'

B. Primers for quantitative genomic PCR.

Bcl11b locus, first round:

5'-GGCTGAATTTACAGGATGAGG-3'

5'-ACTGGAGTTTCCGATGGCC-3'

Bcl11b locus, second round:

5'-GTTTGAGCTTGGAATGGCTGC-3'

5'-ACATCGCCACCATGGAAGAC-3'

Notch1 locus, first round:

5'-CTCCTGCTGCTCGTCCCACTTCCAA-3'

5'-TCCCAGTCAGGGTGGATCCCTCTG-3'

Notch1 locus, second round:

5'-CCATGGTGGAATGCTTTGTATGAGGC-3'

5'-CCCTCAATTTCTCAGGTTCCTTTGAG-3'
